# Impact of an online mindfulness-based program on wellbeing and trait mindfulness for research postgraduate students: a randomized-controlled trial

**DOI:** 10.1186/s40359-024-02233-3

**Published:** 2025-01-10

**Authors:** Jia-Qi Xu, Yee-Man Jennifer Tang, Hoi Ying Katherine Chen

**Affiliations:** 1 Department of Psychology, The University of Hong Kong, Hong Kong, China; 2 Department of Educational Psychology, Chinese University of Hong Kong, Hong Kong, China; 3 English Language Centre, University of Macau, Macau, China

**Keywords:** Wellbeing, Resilience, Wellbeing literacy, Stress, Mindfulness

## Abstract

**Objectives:**

Pursuing a research postgraduate (RPg) degree is a major life event and could be stressful. The current study aims to explore the effectiveness of an online eight-week mindfulness-based intervention on improving wellbeing and alleviating illbeing among a group of RPg students using a randomized waitlist-controlled design.

**Methods:**

A total of 88 RPg students, either studying in Hong Kong or Macau, were recruited (43 were randomized into the immediate intervention group; 67 females; mean age = 27.7; SD = 4.60). The “Finding Peace in a Frantic World” was adopted as the mindfulness-based program. Linear mixed models were applied to test the effects of the intervention on trait mindfulness, compassion, wellbeing related measures (i.e., subjective wellbeing, resilience, wellbeing literacy) and illbeing related measures (i.e., emotional and sleeping disturbances), while controlling for the effects of age and gender. The intervention group was also assessed at two-month follow-up to evaluate the sustained effects.

**Results:**

The results revealed that participants from the intervention group showed increased resilience (*b* = 0.88, *p* = .012), wellbeing literacy (*b* = 2.52, *p* = .04), trait mindfulness (*b* = 5.16, *p* = .006), and decreased emotional disturbances (*b*=-8.24, *p* = .015), while there were no changes in subjective wellbeing, sleeping quality, and self-compassion or compassion towards others compared to the waitlist controls. Positive effects were sustained after two months in the intervention group.

**Conclusions:**

This study provides evidence for online mindfulness training in alleviating RPg students’ emotional disturbance and supporting their resilience and wellbeing literacy.

**Registration:**

The study was retrospectively registered with the ISRCTN Registry on 11/11/2022 (registration number: ISRCTN18262344).

## Introduction

Research postgraduate (RPg) students (i.e., Master of Philosophy [MPhil] or Doctor of Philosophy [PhD] candidates), whose training mainly focus on academic research, face numerous stressors throughout the study period that can induce psychological distress [1, 2]. High levels of mental ill-health (e.g., anxiety, depression, insomnia and suicidal behaviours), alongside low wellbeing and resilience were prevalent among them [3, 4]. Hong Kong and Macau are both special administrative regions, which operate under China’s “one country, two systems” framework. Both regions attract approximately 70% of their doctoral students from mainland China [2]. A qualitative analysis of doctoral students in mainland China, Hong Kong and Macau revealed that pressure of research output and intense competition has caused them enormous stress [2]. In Belgium, half of the RPg students reported psychological distress with one third at risk of common psychiatric disorders, mostly depression [5]. During the COVID pandemic, an online survey in the United Kingdom found around 30% of RPg students met thresholds for probable depression or anxiety [6]. Another study conducted in Hong Kong observed poor sleep hygiene, elevated levels of anxiety and perceived stress among RPg students [7]. These alarming findings highlight a pressing need for effective intervention to enhance wellbeing and alleviate distress among RPg students.

Mindfulness is characterized as purposefully paying attention to experiences in the present moment with openness and acceptance [8, 9]. It is also a trainable human capacity to bring kind awareness in one’s own everyday activities so as to cultivate compassion and wisdom [10]. Through mindfulness training, people learn to alleviate suffering and support wellbeing through engaging in mindfulness practices and integrate acceptance, curiosity, and friendliness in daily activities [11]. A number of mindfulness-based programs (MBPs) have been developed and shown effective across various populations in recent decades [11, 12]. The learning activities of MBPs include didactic teaching (e.g., Walking Down the Street exercise in Mindfulness-Based Cognitive Therapy; MBCT), meditation practices and investigative inquiry between teacher and participants in better understanding their direct experiences derived from mindfulness practices [11].

The curriculum “Finding Peace in a Frantic World” (FPFW) serves as an introductory MBP designed to make mindfulness practices accessible and relevant for the general population in their everyday lives [10]. It is built on the core elements of MBCT, which synergistically combines the practices of mindfulness with the structured methodologies of contemporary Cognitive Therapy (CT) [13]. In weekly sessions, participants can learn about their habitual reactivity patterns by observing the interplay between bodily sensations, thoughts, emotions, and behaviors by engaging in interactive CT exercises in a group setting. Meanwhile, these CT elements are blended with mindfulness training over eight weeks which allows the participants to potentially be more aware of their habitual mind patterns, leading to more skillful responses in their daily lives. Compared to the traditional MBCT curriculum (i.e., 120 to 150 min per session), FPFW is regarded as a less intensive (i.e., 75 to 90 min per session) MBP with shorter duration in mindfulness practices (i.e., 10–15 min). The high-level of commitment of the traditional Mindfulness-Based Stress Reduction (MBSR) or MBCT programs could be a barrier for those who may benefit from mindfulness practice and yet brief mindfulness interventions (e.g., one-off session) may not be adequate to produce positive outcomes [14]. The FPPW program was deemed suitable for RPg students who commonly have a busy schedule.

### Effectiveness of MBPs in alleviating illbeing and enhancing wellbeing

Evidence indicates that MBPs have beneficial impacts on reducing stress and mood disturbances of younger college students in higher education but less research is done on RPG students [15, 16]. Using the FPFW program [10], greater reductions in anxiety but not depression was noticed among university students [17]. With a mixed sample of undergraduate and postgraduate students, researchers found a decrease in mood symptoms using the MBCT program [18–20]. While fewer studies explored effects of mindfulness training on subjective psychological wellbeing, small effect sizes were observed [21, 22]. Helping professional trainees experienced enhanced subjective wellbeing after MBSR program [23]. Mindfulness is also linked to resilience, though evidence is mixed [21].

Wellbeing literacy refers to a malleable capacity to intentionally manage language use and knowledge of wellbeing [24]. The current study takes the initiative to investigate the change of wellbeing literacy as a outcome measure after mindfulness training. The use of language in MBPs has its unique features compared to other psychological interventions. Apart from the teachers’ language in guided meditations, use of turn-taking conversations between the mindfulness teacher and participants, which is known as the inquiry processes after each formal mediation practices, is as an essential vehicle of change in the pedagogical component in MBPs [25, 26]. The way we perceived an experience is shaped by our minds when we describe it in language [27], yet changes in language use after MBPs are rarely studied. This study explores whether attending MBPs affects individuals’ ability to express and understand wellbeing-related language. During the inquiry process in each MBP sessions, teachers guide participants to discuss their direct experiences and reward their noticing with positive feedback [26]. Through continuous practices, positive reinforcement and teacher modelling, the capacity of verbally communicating wellbeing may be enhanced implicitly.

### Mindfulness and compassion

Researchers have identified change of trait mindfulness and self-compassion experienced by the participants during and after the mindfulness training [28–31], which plays an important role in promoting mental health [32, 33].

Compassion, the ability to recognize suffering and motivating action to ease it, is associated with greater wellbeing, higher level of resilience and fewer depressive symptoms [34, 35]. A longitudinal study found that compassion towards self and others predicted lower loneliness and better mental wellbeing [36]. Recognizing common human suffering in oneself and others with a compassionate attitude would be repeatedly observed and cultivated implicitly through the inquiries and group processes [11, 25]. James and Rimes [18] found that self-compassion was significantly higher in the MBCT group compared to the self-help group, which also mediated the group differences in changes in clinical perfectionism.

### Current study

This study aims to evaluate the effectiveness of an eight-week online mindfulness program in improving wellbeing and reducing illbeing among RPg students using a standardized group-based mindfulness program (i.e., “FPFW”). In viewing of the literature on the effectiveness of MBPs on individuals’ mental health as mentioned earlier, both wellbeing and illbeing measures were included in understanding the effectiveness of FPFW among RPg students. For a purpose of explorative investigating change of participants’ language use in MBPs, the self-rated questionnaire for wellbeing literacy [37] was also utilized. It was hypothesized that compared to the waitlist controls, the immediate intervention group would show higher levels of wellbeing (i.e., subjective wellbeing, resilience, and wellbeing literacy), lower levels of illbeing (i.e., emotional and sleeping disturbances), improved trait mindfulness, and enhanced compassion towards oneself and others. It was also hypothesized that the positive effects would be sustained two months post-intervention.

## Materials and methods

### Participants and procedures

Ethics approval was acquired from the Human Research Ethics Committee at University of Hong Kong (EA220260). The current randomized controlled trial (RCT) was registered retrospectively (ISRCTN18262344). Participants were invited to join the research via mass email, posters and other media platforms with the inclusion criteria of (1) current RPg students in any Universities in Hong Kong or Macau, and (2) above 18 years old. To ensure the safety of participating in an online mindfulness program where the teachers cannot immediately reach participants for support, a screening process (i.e., online questionnaire) was conducted to exclude the participants with certain criteria: (1) a history of severe mental illness by self-report (i.e., schizophrenia-spectrum disorder, bipolar disorder, depression, substance abuse); and (2) currently experiencing active psychotic symptoms, high suicidal risk, or a recent personal crisis (e.g., experiencing a severely traumatic event or bereavement).

The promotion and recruitment took place for around one month from mid-June to mid-July 2022. The screening and randomization lasted for around two weeks from mid-July till the end of July 2022. The two classes were conducted from August to September 2022 for the immediate intervention groups while the two classes for the control group were carried out from October to November 2022.

**Fig. 1:**
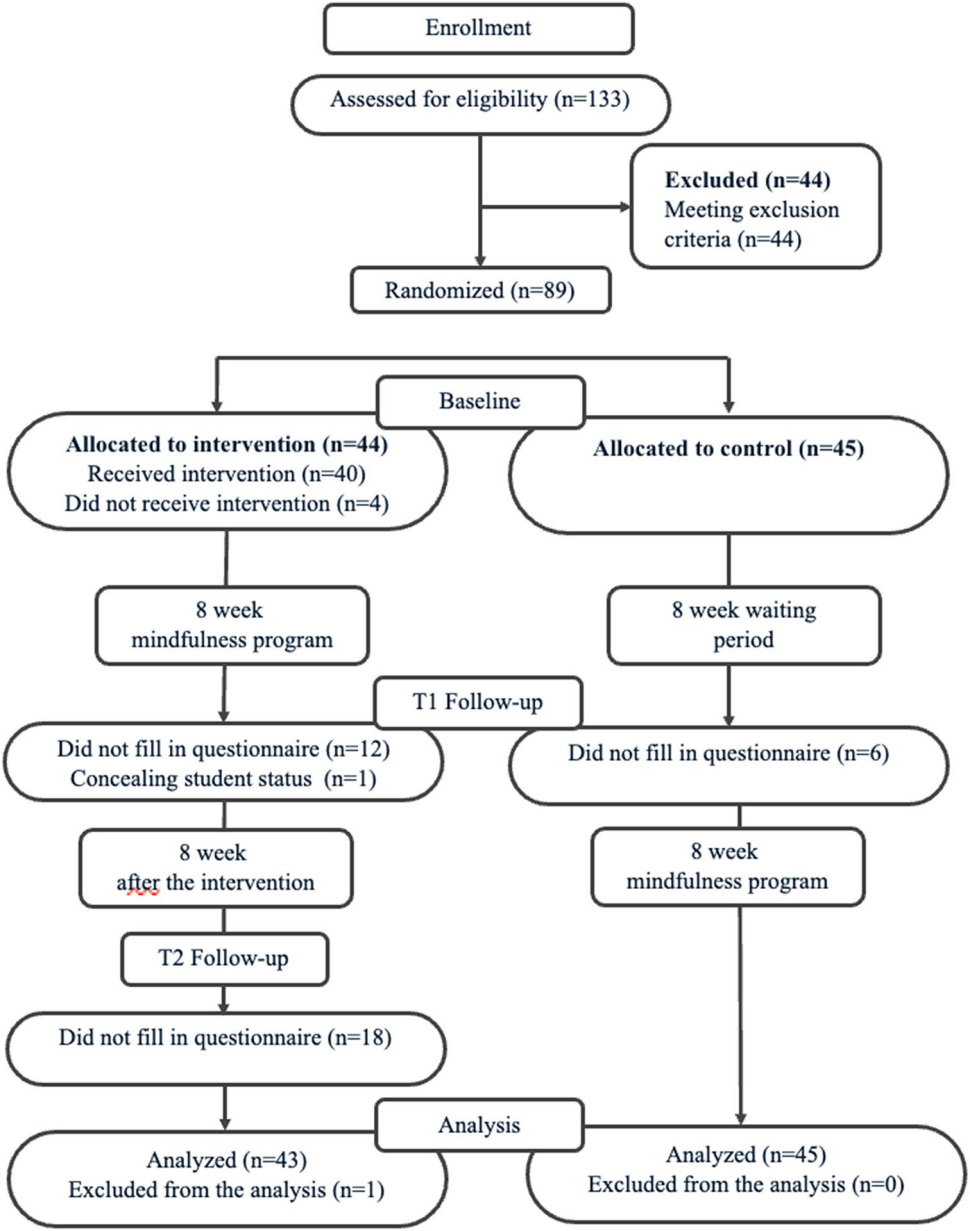
Participants flowchart

A total of 133 students were screened and 44 were excluded due to the exclusion criteria. Eighty-nine participants (67 females, 19 males, two refused to answer) were recruited and randomized (1:1) using computerized randomization formula into the immediate intervention condition or the waitlist control condition (i.e., two months later). One student from the intervention group was later excluded from the analysis as he revealed that he was not a graduate student after intervention. This resulted in a total of 88 participants in the analyses (see Fig. 1). All the participants are Chinese with either Mandarin or Cantonese as their first language. Among all the participants, 25 of them studied either a Mphil or PhD program in Macau, while 63 studied in Hong Kong. No gender difference was observed across the two groups using chi-square test (χ^2^ = 0.89, *p* = .64). T-tests showed no differences in terms of participants’ age (*p* = .089) and baseline measures (*p* > .1) between students in Macau and in Hong Kong.

The consent forms and questionnaire were distributed online with a password protection. Informed consents were obtained from all the participants prior to the data collection. Participants completed the questionnaires online (approximately 10–15 min) at each timepoint. Both groups were assessed at baseline and when the intervention group completed mindfulness training. To evaluate the sustainability of the intervention effects, the intervention group was also assessed two months after intervention.

### Intervention

The mindfulness training program was adapted from the book *Mindfulness: A Practical Guide to Finding Peace in a Frantic World* [10]. The eight-week FPFW program was designed to gradually gain the understanding and skills of recognizing their mind pattern, returning to the present moments, opening up to joy and contentment, responding skilfully with difficulties, and keeping balance amidst ups and downs in life [38]. Compassion for oneself and others are not explicitly taught in FPFW but it is implicitly incorporated into some mindfulness practices (e.g., befriending meditation and exploring difficulty meditation). Compared with the traditional MBCT, FPFW is shorter in session duration and without the one-day silent retreat. In the current study, each weekly session was 90 min which included mindfulness practices, inquiry, interactive group exercises based on CT, and assigning daily home practices (i.e., around 20 min per day). The mindfulness practices in FPFW are around 10–15 min. In weekly assigned home practices, except mindfulness practices, FPFW curriculum also encourages integrating mindfulness into daily life so as to develop new positive habits in life such as “random acts of kindness”. The courses were delivered online via Zoom (http://zoom.us/).

Two classes for each group were delivered in parallel with a mix of participants from Hong Kong and Macau by the two trained mindfulness teachers (i.e., the first and last authors) who completed one-year Teacher Training Programme with the Oxford Mindfulness Foundation. Both mindfulness teachers speak fluent English, Mandarin, and Cantonese. To accommodate the language preferences of the participants, one of the parallel classes was delivered in Mandarin while the other was in English. During the course, the two teachers met regularly with their supervisors to ensure the intervention integrity based on the “The Mindfulness-Based Interventions: Teaching and Learning Companion (MBI: TLC)” framework [39]. A self-developed website and shared folders were provided for course information and meditation soundtracks. Participants were encouraged to share written practice reflections with teachers to support their practices.

### Measurements

Participants’ demographic (i.e., age and gender) and background information, i.e., mode of study (i.e., fulltime or parttime), year of study, and curriculum (i.e., PhD or MPhil), University location, prior experience with mindfulness training and mindfulness practices were collected. Trait mindfulness, compassion for self and others, wellbeing and illbeing were also assessed via self-rated questionnaires. The fidelity of the mindfulness intervention had been ensured at three layers: (1) the two teachers shared lesson plans that adhered to the teaching manual and were under regular supervision using “MBI: TLC” [39] framework throughout the whole teaching period; (2) after teaching, one student helper also checked whether all the activities outlined on the lesson plans were carried out by listening to the audio recordings of each session; (3) feedback from the participants were also collected. They were asked to rate on “how much they have benefited from the course” and “how is the teaching quality” right after the intervention on a 10-point Likert scale.

Three items on participants’ home practices qualities (i.e., “number of formal meditation practice per week”, “average duration in minutes of each formal meditation practice”, and “number of times of applying mindfulness in daily life per week”) were collected both immediately after the intervention and at the two-month follow-up.

### Wellbeing measures

The Short Warwick-Edinburgh Mental Well-being Scale (SWEMWBS) is a seven-item questionnaire to measure the subjective wellbeing with a 5-point Likert scale [40]. The Chinese version was validated among the general population in Hong Kong [41]. A total score was calculated to indicate level of well-being. Cronbach’s alpha coefficients of the scale at baseline (T0), right after intervention (T1), and at two-month follow-up (T2) were 0.82, 0.81, and 0.92.

The Well-being Literacy Scale is a six-item instrument to measure the capability to comprehend and communicate wellbeing [24]. The original scale was developed and validated across different population in Australia [37]. The Chinese version of the scale was provided and permitted for use by the first author of the original paper [37]. A total score was calculated to indicate capacity of wellbeing language use. Cronbach’s alpha coefficients of the scale at T0, T1 and T2 were 0.90, 0.90, and 0.96.

The Connor-Davidson Resilience Scale – 2 items (CD-RISC2) is a short version of the Connor-Davidson Resilience Scale to measure individual’s resilience [42, 43]. The Chinese version was translated and validated among general population in Hong Kong [44]. A total score was computed to indicate level of resilience. Cronbach’s alpha coefficients of the scale at T0, T1 and T2 were 0.78, 0.77, and 0.88.

### Illbeing measures

Depression, Anxiety and Stress Scale – 21 items (DASS-21) measures emotional states of depression, anxiety, and stress (Lovibond & Lovibond, 1995). The Chinese version was validated by Moussa et al. [45]. The total score was calculated to reflect overall emotion disturbance with a higher score indicating higher level of emotion disturbance. The subscales were also used in the analyses. Cronbach’s alpha coefficients of the scale at T0, T1 and T2 were 0.90, 0.89, and 0.85.

Sleeping quality were measured using the 19-item Pittsburgh Sleep Quality Index [46], which form seven components. A global score was computed by aggregating seven components’ scores, indicating the overall sleeping quality with a higher score implying a poorer sleeping quality. The Chinese version of the scale was validated in undergraduate students with the Cronbach’s alpha of 0.66 [47]. Cronbach’s alpha coefficients among the seven components of the scale in the current study were 0.64, 0.44, and 0.67 at T0, T1 and T2.

### Mindfulness

To assess the general mindfulness awareness, the 15-item Five Facet Mindfulness Questionnaire (FFMQ-15) was used to measure trait mindfulness [48]. The translated version used in the current study was validated in a Taiwanese population [49]. Five facets of trait mindfulness were derived from the factor analysis, including observing (i.e., propensity to observe internal and external experience), describing (i.e., describe inner experiences with words), acting with awareness (i.e., act with awareness of the present), nonjudging (i.e., take a non-judgemental attitude towards one’s own experience), and nonreactivity (i.e., let one’s thoughts and feelings go without focusing or elaborating on them) [50]. The total score of trait mindfulness and the five facets sub-scores were adopted in the analyses. Cronbach’s alpha coefficients of the whole scale at T0, T1 and T2 were 0.80, 0.80, and 0.88 respectively.

### Compassion

The Sussex-Oxford Compassion Scales are self-report measures of self-compassion and compassion for others, which composed of 20 items each for Sussex-Oxford Compassion for Others Scale (SOCS-O) and Sussex-Oxford Compassion for the Self Scale (SOCS-S) [34]. Two total scores of both scales were calculated to indicate the level of compassion towards oneself and for others respectively. Cronbach’s alpha coefficients for SOCS-O at T0, T1 and T2 were 0.95, 0.95, 0.92, while 0.96, 0.97, and 0.96 for SOCS-S.

### Statistical analyses

Statistical analyses were conducted using Stata 17 (Stata Corp., College Station, TX, USA). The baseline characteristics were compared using t-tests and chi-squared tests. Linear mixed models tested the interactions of time and group controlling for the effects of age and gender. Linear mixed models also evaluated the sustained effects from the intervention by comparing the changes of the measures at baseline (T0), immediately after (T1) and two months after (T2) mindfulness training in the intervention group. Analyses were first conducted using an intention-to-treat (ITT) sample, including data from all participants (*N* = 88). Per protocol analyses, including those who filled in the questionnaires across timepoints and attended at least half of the sessions (i.e., 4 or more), were provided as supplementary information. To explore the effect of home practice on outcome measures, linear regression models were conducted using the three items of home practice qualities (see “Measurements”) as independent variables while controlling for the effects of age and gender. We set the significance level at 0.05, without further adjustment of multiple comparisons as the current study is exploratory in nature [51].

## Results

No group differences were found in demographic features and baseline measures (see Table 1). Eight out of 88 participants indicated that they had past experiences with mindfulness training. One of them took a 8-week MBCT program, one had previous experience of 10-days silent retreat, and the others took one-off mindfulness workshops. Fourteen of them indicated practicing mindfulness regularly before the course. Chi-square tests showed no difference was observed between the intervention and control groups in terms of the number of participants with prior mindfulness training experience or mindfulness practice (χ^2^ = 2.01, *p* = .16; χ^2^ = 0.24, *p* = .62). T-tests indicated that no significant differences were noted in all the baseline measures between individuals with or without previous training, or between those with or without prior mindfulness practices (all *p* > .11).

**Table 1:** Participants’ descriptive statistics and baseline measures

	Control (*N* = 45)	Intervention (*N* = 43)	Differences (t/χ^2^, *p* value)
Variable/Measure	Mean (SD)/Ratio		
Age	28.42 (0.71)	26.74 (0.66)	t = 1.73, *p* = .09
Gender (M: F:Unknow)	10:32:1	9:35:1	χ^2^ = 0.14, *p* = .93
Curriculum (PhD: MPhil)	22:21	24:21	χ^2^ = 0.04, *p* = .84
Mode of learning (PT: FT)	41:2	38:7	χ^2^ = 2.85, *p* = .09
Year (1:2:3:4:5)	20:12:4:6:1	20:14:5:5:1	χ^2^ = 31, *p* = .99
Trait Mindfulness (total)	48.40 (7.02)	48.19 (8.72)	t = 0.13, *p* = .90
*Observing*	10.00 (2.95)	9.70 (3.04)	t = 0.47, *p* = .64
*Describing*	11.00 (2.02)	10.42 (2.35)	t = 1.24, *p* = .22
*Acting with awareness*	9.69 (2.11)	10.49 (2.57)	t=-1.60, *p* = .11
*Nonjudging*	9.84 (2.28)	9.63 (2.78)	t = 0.41, *p* = .69
*Nonreactivity*	7.87 (2.11)	7.95 (2.43)	t=-0.18, *p* = .86
Compassion (self)	69.96 (13.66)	73.21 (13.94)	t=-1.11, *p* = .27
Compassion (others)	74.62 (13.07)	75.65 (11.64)	t=-0.39, *p* = .70
Subjective wellbeing	23.2 (3.71)	23.35 (4.17)	t=-0.27, *p* = .86
Wellbeing literacy	30.89 (4.59)	30.37 (5.72)	t = 0.47, *p* = .64
Resilience	5.38 (1.29)	5.05 (1.48)	t = 1.12, *p* = .26
Emotional disturbances	21.56 (16.57)	22.74 (16.02)	t=-0.34, *p* = .73
*Stress*	9.69 (0.99)	11.35 (1.28)	t=-1.03, *p* = .31
*Anxiety*	5.91 (0.91)	5.77 (0.58)	t = 0.12, *p* = .90
*Depression*	5.96 (0.83)	5.63 (0.84)	t = 0.28, *p* = .78
Sleep quality	6.76 (2.90)	6.23 (2.46)	t = 0.91, *p* = .37

In the intervention group, 34 out of 43 participants (79%) joined at least half of the mindfulness training sessions. T-tests and chi-squared tests showed no significant differences in demographic characteristics between those who joined at least half of the program and those who did not. However, the participants who jointed at least half sessions had lower scores in the facet of “acting with awareness” in trait mindfulness (t = 2.23, *p* = .031), higher scores in stress subscale (t=-2.14, *p* = .038), anxiety subscale (t=-2.56, *p* = .014), and total score of emotional disturbance (i.e., DASS-21; t=-2.44, *p* = .019) at the baseline.

Table 2 shows participants’ outcomes in the intervention group after the mindfulness program compared with the controls. The assumptions of all linear mixed models for both the ITT and per protocol samples were assessed. The Kolmogorov-Smirnov tests were used to examine the normality of residuals. The results indicated that the residuals were normally distributed (*p* > .055) for both the ITT sample and per protocol sample, with the exception of the scores of compassion for others in the ITT sample (*p* = .036). Violation of distributional assumptions does not always cause significant bias in the results of mixed models as long as the distribution is unimodal [52]. With a skewness of − 0.93, the scores of compassion for others in the ITT sample exhibited a negatively skewed unimodal distribution, and hence the use of linear mixed model was considered a reliable method for analysis.

**Table 2:** Mixed models for intervention effect (group x time) controlled with age and gender

	Intention-to-treat (*N* = 88)			Per-protocol (*N* = 70)		
Measures	coefficient	*p value*	95% CI	coefficient	*p value*	95% CI
Trait Mindfulness	5.16	0.006	(1.44, 8.88)	5.95	0.002	(2.09, 9.80)
*Observing*	1.11	0.12	(-0.30, 2.52)	1.20	0.108	(-0.26, 2.66)
*Describing*	1.22	0.012	(0.27, 2.16)	1.25	0.011	(0.29, 2.21)
*Acting with awareness*	0.10	0.86	(-1.04, 1.25)	0.60	0.306	(-0.55, 1.75)
*Nonjudging*	1.57	0.006	(0.44, 2.69)	1.72	0.003	(0.60, 2.85)
*Nonreactivity*	1.21	0.049	(0.007, 2.41)	1.18	0.068	(-0.09, 2.44)
Compassion (self)	3.01	0.38	(-3.76, 9.79)	4.28	0.231	(-2.73, 11.30)
Compassion (others)	2.89	0.38	(-3.59, 9.37)	3.16	0.34	(-3.39, 9.72)
Subjective wellbeing	1.11	0.23	(-0.70, 2.92)	1.37	0.135	(-0.43, 3.18)
Wellbeing literacy	2.52	0.04	(0.11, 4.93)	2.77	0.028	(0.30, 5.23)
Resilience	0.88	0.012	(0.19, 1.57)	0.98	0.006	(0.28, 1.68)
Emotional disturbances	-8.24	0.015	(-14.88, -1.60)	-10.36	0.002	(-16.95, -3.77)
*Stress*	-4.51	0.010	(-7.97, -1.06)	-5.50	0.002	(-9.02, -2.00)
*Anxiety*	-1.57	0.230	(-4.13, 0.99)	-2.75	0.031	(-5.25, − 0.26)
*Depression*	-1.76	0.067	(-3.64, 0.12)	-2.10	0.028	(-3.97, − 0.22)
Sleep quality	− 0.74	0.30	(-2.13, 0.65)	− 0.86	0.237	(-2.29, 0.57)

In the ITT sample, no missing data was observed at the baseline. The percentage of missing data at T1 (after intervention) was 20.45%. At two-month follow-up, 58.14% of the participants in the intervention group filled in the questionnaire. Linear mixed models adopted in the analyses are robust to various types of missing data, including missing at random missing completely at random, and not missing at random, thereby allowing for some missing data at certain time points [53]. Furthermore, we analyzed both ITT and per-protocol samples, with the per-protocol sample including data only from participants who responded to questionnaires at all timepoints for both groups and attended half or more program sessions in the intervention group. The interquartile range (IQR) method and the Z-score method were employed to identify outliers in both the intention-to-treat and per-protocol samples. Given that the percentage of outliers was less than 8% across all datasets, all data were retained to avoid the potential impact of arbitrary data removal on the overall results [54].

### Intention-to-treat analyses

#### Changes after the mindfulness program

The linear mixed models (see Table 2) showed a significant interaction between group and time after the mindfulness program in trait mindfulness as reflected by the FFMQ-15 total score (*b* = 5.16, *p* = .006). Significant interactions were also found in terms of “describing” (*b* = 1.11, *p* = .012), “nonjudging” (*b* = 1.57, *p* = .006), and “non-reactivity” (*b* = 1.21, *p* = .049), but not in “observing” (*b* = 1.11, *p* = .12) and “acting with awareness” (*b* = 0.10, *p* = .86). It indicates that the overall trait mindfulness was significantly improved immediately after the mindfulness training, in particular in the facets of “describing”, “nonjudging”, and “non-reactivity”. No significant interactions were found in term of both compassion for oneself (*b* = 3.01, *p* = .38) and others (*b* = 2.89, *p* = .38).

In terms of wellbeing, the linear mixed models between group and time demonstrated significant interactions in terms of wellbeing literacy (*b* = 2.52, *p* = .04) and resilience (*b* = 0.88, *p* = .012), implying the intervention group showed significant improvements in wellbeing literacy and resilience after the mindfulness program compared to the controls. No significant interaction was found in subjective wellbeing (*b* = 1.11, *p* = .23).

In terms of illbeing, the interaction between group and time in general emotional disturbance as reflected by the DASS total score was significant (*b*=-8.24, *p* = .015), indicating participants’ emotional disturbance significantly dropped after the mindfulness program as compared to the controls. However, only the stress subscale exhibited a significant interaction (*b*=-4.51, *p* = .010). The interaction in the overall sleep quality was not significant (*b*=-0.74, *p* = .30).

### Changes at two-month follow-up

Linear mixed models were conducted in the intervention group controlling age and gender at baseline (T0), immediately after (T1), and two-months after the mindfulness training (T2) to evaluate the sustained intervention effects. Contrasts based on the linear mixed models were reported as to show the pairwise comparisons between T1 and T0, T2 and T0, as well as T2 and T1 (see Table 3).

**Table 3:** Contrasts for the longitudinal changes in the intervention group

Measures									
**Intention-to-treat** (*N* = 43)	T1 v T0			T2 v T0			T2 v T1		
	Contrast	*p*	95% CI	Contrast	*p*	95% CI	Contrast	*p*	95% CI
Trait Mindfulness	5.34	< 0.001	2.65, 8.04	4.92	< 0.001	2.01, 7.84	− 0.42	0.784	-3.42, 2.59
*Observing*	0.76	0.125	− 0.21, 1.73	0.721	0.177	− 0.33, 1.77	− 0.04	0.947	-1.12, 1.05
*Describing*	0.88	0.010	0.21, 1.55	0.90	0.016	0.17, 1.62	0.01	0.971	− 0.73, 0.76
*Acting with awareness*	0.29	0.501	− 0.56, 1.15	0.35	0.454	− 0.57, 1.28	0.06	0.904	− 0.90, 1.02
*Nonjudging*	1.91	< 0.001	1.11, 2.71	1.17	0.008	0.30, 2.03	− 0.74	0.100	-1.63, 0.14
*Nonreactivity*	1.52	0.001	0.59, 2.45	1.78	< 0.001	0.78, 2.78	0.26	0.621	− 0.79, 1.32
Compassion (self)	3.31	0.163	-1.34, 7.95	5.50	0.032	0.48, 10.53	2.20	0.408	-3.00, 7.40
Compassion (others)	1.13	0.63	-3.48, 5.74	3.28	0.197	-1.70, 8.25	2.14	0.418	-3.04, 7.32
Subjective wellbeing	0.87	0.192	− 0.44, 2.18	0.95	0.302	− 0.47, 2.37	0.08	0.915	-1.38, 1.54
Wellbeing literacy	2.69	0.004	0.86, 4.52	3.35	< 0.001	1.37, 5.32	0.66	0.529	-1.39, 2.70
Resilience	2.36	< 0.001	1.81, 2.91	2.53	< 0.001	1.94, 3.13	0.18	0.575	− 0.44, 0.80
Emotional disturbances	-4.34	0.127	-9.92, 1.24	-5.41	0.078	-11.42, 0.60	-1.07	0.739	-7.38, 5.24
*Stress*	-2.39	0.111	-5.33, − 0.55	-2.73	0.090	-5.88, 0.43	− 0.34	0.841	-3.68, 2.99
*Anxiety*	− 0.82	0.390	-2.69, 1.05	-1.93	0.059	-3.94, 0.07	-1.11	0.304	-3.24, 1.01
*Depression*	-1.10	0.137	-2.54, 0.35	− 0.56	0.481	-2.13, 1.00	0.53	0.514	-1.07, 2.14
Sleep quality	− 0.53	0.225	-1.40, 0.33	− 0.70	0.141	-1.63, 0.23	− 0.17	0.737	-1.13, 0.80
**Per-protocol** (*N* = 25)	T1 v T0			T2 v T0			T2 v T1		
	Contrast	*p*	95% CI	Contrast	*p*	95% CI	Contrast	*p*	95% CI
Trait Mindfulness	5.32	0.001	2.08, 8.56	4.72	0.004	1.48, 7.96	− 0.60	0.717	-3.84, 2.64
*Observing*	0.52	0.389	− 0.66, 1.70	0.52	0.389	− 0.66, 1.70	0.00	1.00	-1.18, 1.18
*Describing*	0.84	0.035	0.06, 1.62	0.80	0.045	0.02, 1.58	− 0.04	0.920	− 0.82, 0.74
*Acting with awareness*	0.32	0.511	− 0.63, 1.27	0.44	0.366	− 0.51, 1.39	0.12	0.805	− 0.83, 1.07
*Nonjudging*	2.52	< 0.001	1.69, 3.35	1.44	< 0.001	0.61, 2.27	-1.08	0.011	-1.91, − 0.25
*Nonreactivity*	1.12	0.049	0.01, 2.23	1.52	0.007	0.41, 2.63	0.40	0.481	− 0.71, 1.51
Compassion (self)	2.00	0.488	-3.65, 7.65	4.48	0.120	-1.17, 10.13	2.48	0.390	-3.17, 8.13
Compassion (others)	0.96	0.728	-4.44, 6.36	2.56	0.353	-2.84, 7.96	1.60	0.562	-3.80, 7.00
Subjective wellbeing	1.00	0.198	− 0.52,2.52	0.92	0.237	− 0.60, 2.44	− 0.08	0.918	-1.60, 1.44
Wellbeing literacy	3.04	0.004	0.96, 5.12	3.52	< 0.001	1.44, 5.60	0.48	0.652	-1.60, 2.56
Resilience	2.32	< 0.001	1.68, 2.96	2.48	< 0.001	1.84, 3.11	0.16	0.623	− 0.48, 0.80
Emotional disturbances	-10.00	0.001	-15.95, -4.05	-8.48	0.005	-14.43, -2.53	1.52	0.616	-4.43, 7.47
*Stress*	-4.96	0.003	-8.26, -1.66	-4.40	0.009	-7.70, -1.10	0.56	0.740	-2.74, 3.86
*Anxiety*	-3.20	0.002	-5.22, -1.18	-3.20	0.002	-5.22, -1.18	0.00	1.00	-2.02, 2.02
*Depression*	-1.84	0.026	-3.46, − 0.22	− 0.88	0.287	-2.50, 0.74	0.96	0.246	− 0.66, 2.58
Sleep quality	− 0.12	0.796	-1.03, 0.788	− 0.32	0.490	-1.23, 0.588	− 0.20	0.666	-1.11, 0.71

Note T0 = baseline; T1 = after 8-week intervention; T2 = 8 week after the intervention

Participants’ trait mindfulness significantly improved from T0 to T1 [Contrast = 5.34, *p* < .001, 95%CI (2.65, 8.04)] and T0 to T2 [Contrast = 4.92, *p* < .001, 95%CI (2.01, 7.84)]. Pertaining to five facets, significant effects of time from T0 to T1 and T0 to T2 were found in “describing”, “nonjudging”, and “nonreactivity” (see Table 3). Significant changes were found in “describing” from T0 to T1 [Contrast = 0.88, *p* = .01, 95%CI (0.21, 1.55)], and T2 to T0 [Contrast = 0.90, *p* < .001, 95%CI (0.17, 1.62)]. Changes of “nonjudging” from T0 to T1 [Contrast = 1.91, *p* < .001, 95%CI (1.11, 2.71)] and T0 to T2 [Contrast = 1.17, *p* = .008, 95%CI (0.30, 2.03)]; and “nonreactivity”, from T0 to T1 [Contrast = 1.52, *p* = .001, 95%CI (0.59, 2.45)] and T0 to T2 [Contrast = 1.78, *p* < .001, 95%CI (0.78, 2.78)] were also observed. No significant results were found in both compassion scales.

Regarding wellbeing measures, resilience was significantly improved from T0 to T1 [Contrast = 2.69, *p* = .004, 95%CI (0.86, 4.52), and T0 to T2 [Contrast = 3.35, *p* < .001, 95%CI (1.37, 5.32)]. Significant time effects were also found in wellbeing literacy from T0 to T1 [Contrast = 2.36, *p* < .001, 95%CI (1.81, 2.91)], and T0 to T2 [Contrast = 2.53, *p* < .001, 95%CI (1.94, 3.13)]. No significant effect of time on subjective wellbeing was found.

Regarding ill-being measures, no significant effects of time was found in terms of the DASS total score, the three DASS subscales and the overall sleeping quality. ITT analyses showed no significant differences found in all the measures from T1 to T2 (see Table 3) in the intervention group.

### Per protocol analyses

Per protocol analyses were conducted for all the measures (see Tables 2 and 3). Thirty-one in the intervention group attended half or more program sessions and filled in the questionnaires at T0 and T1 while 39 in the control group filled in the questionnaire at both timepoints. Seventy participants were thus included in the per protocol analyses to evaluate the changes after the mindfulness program. No significant differences were found in the demographic characteristics and baseline measure between those included (*N* = 70) and excluded (*N* = 18) in the per protocol analyses. Most results of the ITT analyses were confirmed in the per protocol analyses while only few outcomes were different from the ITT models (see Table 2). In the per protocol analyses, linear mixed models between group and time showed significant interaction in DASS anxiety (*b*=-2.75, *p* = .031) and depression subscales (*b*=-2.10, *p* = .028), which were not significant in the ITT analyses. While significant interaction was found in “non-reactivity” (*b* = 1.21, *p* = .049) using the ITT analyses, the interaction was not significant in the per protocol analysis.

Per protocol analyses were also conducted to investigate the changes at two months follow-up in the intervention group. The 25 participants from the intervention group included in the per protocol analysis showed higher anxiety subscale (t=-2.36, *p* = .023), and stress subscale (t=-2.07, *p* = .045) than those excluded. Most results were similar as those in the ITT analyses except “nonjudging” facet of trait mindfulness and emotional disturbance (see Table 3). The “nonjudging” facet was significantly decreased from T1 to T2 [Contrast=-1.08, *p* = .011, 95%CI (-1.91, − 0.25)], indicating a decrease in non-judgemental attitude two-months after the intervention. Participants’ DASS total scores significantly decreased from T0 to T1 [Contrast=-10.00, *p* = .001, 95%CI (-15.95, -4.05)], and T0 to T2 [Contrast=-8.48, *p* = .005, 95%CI (-14.43, -2.53)]. The stress subscale was significantly decreased from T0 to T1 [Contrast=-4.96, *p* = .003, 95%CI(-8.26, -1.66)], and T0 to T2 [Contrast=-4.40, *p* = .009, 95%CI (-7.70, -1.10)]. The anxiety subscale was decreased from T0 to T1 [Contrast=-3.20, *p* = .002, 95%CI (-5.22, -1.18)], T0 to T2 [Contrast=-3.20, *p* = .002, 95%CI (-5.22, -1.18)]. The depression sub-score was decreased from T0 to T1 [Contrast=-1.84, *p* = .026, 95%CI (-3.46, − 0.22)].

To ensure the fidelity of the mindfulness intervention, feedback from the participants and their home practice qualities were analyzed using per protocol sample. The mean of participants’ rating on “how much you have benefited from the course” is 8.03 (SD = 1.72) out of ten while the mean of their perception of teaching quality was 9.25 (SD = 0.82) out of ten. The helper also checked that all the activities in the eight-week sessions were carried out as planned by two teachers.

During the eight-week course, participants practiced formal meditation (e.g., sitting meditation, body scan) 3.84 times on average (SD = 1.63) and the mean duration of the formal practices was 15.26 min (SD = 5.20). Mean number of times to apply mindfulness in life per week (e.g., noticing the feet on the ground, keeping body in mind while talking) is 5.69 (SD = 4.31). At the follow-up, the participants reported practicing formal meditation for 2.34 times (SD = 2.41) per week with a mean duration of 12.12 min (SD = 8.13). They also applied mindfulness in daily life for 5.76 times (SD = 9.82) after the intervention. Linear regression models were conducted using the outcome measures as dependent variables and the three items of home practice qualities as independent variables while controlling age and gender. Quality of home practices did not predict any of the outcome measures (all *p* > .10) across all the models.

## Discussion

The current study explored the effects of a less intensive MBP (i.e., FPFW) in reducing illbeing and enhancing wellbeing among RPg students with an RCT design. We also investigated intervention effects of trait mindfulness, compassion for others and self-compassion.

The results based on the ITT analyses showed significant intervention effects of FPFW in overall trait mindfulness, wellbeing literacy, resilience, and overall emotional disturbance while no improvement in compassion for oneself or others, subjective wellbeing, and sleeping quality. It indicated that a less intensive mindfulness training (i.e., 90 minutes weekly session over eight weeks) could enhance participants’ resilience and their use of wellbeing language while alleviating their overall psychological distress. The supplementary results based on the per-protocol sample were discussed, particularly when the results differed from the ITT analyses.

### Changes of illbeing and wellbeing after MBP

Existing evidence has demonstrated that MBSR and MBCT were effective in decreasing stress, anxiety and depression symptoms in non-clinical population, with large effect sizes for stress and moderate effects for anxiety, depression and subjective wellbeing [22, 55]. A recent meta-analysis indicated that there is no evidence suggesting that larger doses (i.e., program and session length, frequency, and duration of recommended practice) are more effective than smaller doses in predicting depression, anxiety, and stress outcomes [56]. However, the less intensive version of MBCT, i.e., FPFW, was seldom studied in the existing literature. Simonsson and colleagues [17] found a significant reduction in anxiety but not depression was noticed after the 8-week FPFW program among university students. Likewise, the current study demonstrated an interaction effect of time and group in overall emotional disturbance immediately after the intervention and the effect was maintained after two months. Nevertheless, when looking into the subscales in DASS, we only found a significant decrease in stress level but not in levels of anxiety and depression using ITT sample. On the other hand, the per protocol analyses showed significant reductions in stress, anxiety and depression. In the intervention group, we observed a similar pattern that significant changes in the overall DASS and its three subscales before and after intervention were only observed in the per protocol sample but not the ITT sample. It was found that both baseline anxiety and stress levels were significantly higher in those included than those excluded in the per protocol analyses of the intervention group. It may imply that RPg students experiencing greater emotional distress were more likely to adhere to the MBP, potentially resulting in greater benefits from the mindfulness training.

Meanwhile, the current study did not find significant effects in enhancing sleeping quality and subjective wellbeing. Poor sleep quality and sleeping deprivation are commonly observed among graduate students [1]. Existing evidence has shown positive effects of mindfulness training on improving sleep quality mainly in clinical populations [57, 58]. Consistent with the current finding, one recent published systematic review article found no significant effects MBSR on sleep quality or subjective wellbeing among university students [59]. On the other hand, Querstrets and colleagues [22] found that longer interventions with more training time were associated with increased quality of life or wellbeing in a non-clinical population. The insignificant improvement of subjective wellbeing in the current study could be due to the shorter training duration, lower intensity of training (i.e., shorter formal mindfulness practices) and/or absence of one-day retreat in FPFW. Further investigation is thus needed to compare the effects of less intensive MBP (e.g., FPFW) and a traditional MBP (e.g., MBCT).

In addition to subjective wellbeing, the current study investigated resilience and capacity of well-being literacy after the 8-week mindfulness training. The results showed that participants in the intervention group experienced increased resilience and improved well-being literacy. The positive effects persisted for two months after the intervention. Similarly, MBCT was found to be effective in increasing resilience in patients recovered from COVID-19 [60], indicating mindfulness practice as a protective factor for mental wellbeing under adverse situations [61, 62]. Moreover, the current study took the initiative to investigate the self-report change of language use (i.e., wellbeing literacy) after the FPFW program. Wellbeing literacy is a language-based capacity which allows individuals to intentionally use their vocabulary, knowledge and language skills to promote the wellbeing of oneself, or others [24, 37]. It is a construct that is highly related to but distinct from wellbeing [37]. Although this concept was never introduced or practiced explicitly in the mindfulness sessions, it is speculated that through communicating one’s experience during the conversational interactions in MBPs, the participants had been practicing comprehending and expressing wellbeing language. As a result, their repertoire of knowledge and skills in promoting wellbeing had been expanded while their intention of use the language to improve the wellbeing of oneself and others had been enhanced. An alternative explanation is that wellbeing literacy is improved through other components in the FPFW program (e.g., mindfulness practice or didactic teaching) rather than the inquiry process during the sessions. Future studies could compare the outcomes of self-directed (e.g., use mobile app) and teacher-led mindfulness training to investigate the mechanism of change in wellbeing literacy. However, the positive outcome (*p* = .04 in the ITT sample and *p* = .028 in the per protocol sample) of wellbeing literacy should be interpreted with caution as the current study did not control for multiple comparisons. More confirmatory studies should be conducted to further investigate the effect of MBP in wellbeing literacy. To sum up, resilience and wellbeing literacy, as capacities closely associated with wellbeing, may potentially serve as individuals’ internal resources under challenging situations.

### Mindfulness and compassion after MBP

Overall trait mindfulness had been significantly improved after the mindfulness training. Measuring trait mindfulness using FFMQ allowed us capture both “what” and “how” of the interactive mindfulness process in five distinct domains [63]. After the mindfulness training, the domains of “describing”, “nonjudging”, and “non-reactivity” showed significant improvement, which was sustained even after two months. However, change of “non-reactivity” was only marginally significant (*p* = .049) in the ITT analysis and not significant in the per protocol analysis. The effect of mindfulness training on “non-reactivity” should thus be interpreted with caution. The “describing” domain refers to the ability of labelling inner experiences with words, which is “what” skills in mindfulness training. Meanwhile “nonjudging” towards one’s inner experience refers the “how” skills of mindful attention, which is one of the essential attitudes one adopts in mindfulness practices. Stein and Witkiewitz [12] had recognized both the content (i.e., the mindfulness practices) as well as attitudes of acceptance, openness and nonreactivity to experience as two promising active components in MBPs. The current results demonstrated that at least some of the “what” and “how” skills in mindfulness practices were improved after the 8-week online mindfulness training. Nevertheless, not all the facets of trait mindfulness (i.e., facets of “observing” and “acting with awareness”) were found to be improved after the intervention. Similar findings were observed in other studies with brief mindfulness training, where not all facets showed significant changes, despite a significant improvement in overall trait mindfulness [64, 65]. Future studies need to further explore the pattern of changes in different domains of trait mindfulness in less intensive mindfulness trainings.

Existing literature indicates that MBPs had positive effects in improving participants’ self-compassion with heterogenous effects at a medium to large degree [66]. A meta-analysis also revealed significant therapeutic effects of mindfulness-based interventions on empathy in the healthy population while highlighting the impact of intervention dosage and format (online versus offline) on treatment outcomes [67]. On the contrary, our findings revealed that both self-compassion and compassion for others did not show significant improvement both immediately after the mindfulness training and at follow-up. One possible reason is that compassion for others and self-compassion were not delivered as an explicit theme in the current intervention. In their study comparing the effects of explicit compassion training program and MBSR, Gonzalo Brito-Pons and colleagues found the explicit training of compassion had a greater impact on enhancing compassionate abilities, particularly in terms of empathic concern and recognizing shared humanity [68]. Besides, Wasson and colleagues [69] found enhanced self-compassion among individuals after mindfulness-based interventions and they also discovered that a component of silent retreat in the program showed a significantly larger effect size. Further exploration is needed to investigate the roles of specific meditations, program intensity and retreat in enhancing compassion in MBPs.

### Limitations

Despite the encouraging findings, there are yet some limitations. One of the limitations was the drop-out rate. Seventy out of 88 participants (79.55%) completed both pre- and post-questionnaires whereas 25 out of 43 participants (58.14%) in the intervention group filled in the two-months follow-up questionnaire. Supplementary per protocol analyses were thus presented to confirm the results from the ITT analyses. However, due to the high missing rate in the two-month follow up data, the sustained effects observed in the intervention group shall be interpreted with caution. There could be a survival bias in the follow-up results two months after the mindfulness training, as those who found the program more beneficial might be more likely to participate in the research.

Another limitation is that multiple comparisons were not corrected in the current analyses due to the exploratory nature of the study. Results that are marginally significant should be interpreted with caution (e.g., wellbeing literacy, non-reactivity subscale of trait mindfulness). Confirmatory studies are needed to validate the effects of less intensive mindfulness training especially when utilizing these outcome measures.

The current study took the initiative to explore the change of expressing and comprehending wellbeing language (i.e., wellbeing literacy) after eight week’s mindfulness training. However, the measure of wellbeing language capacity was purely based on self-rating on a Likert-scale questionnaire. Future studies may further include a qualitative approach together with self-rate measures to further investigate the change of the contents in the participants’ discourse before, during, and after the MBP.

### Implications and future directions

It is worthy to note that out of 133 applicants, 44 (33%) were excluded from the study due to a history of severe mental illness or current mental health challenges. Graduate program in the Universities should be aware of their students’ need for prevention or early intervention for mental illness. Results from the current RCT indicated the feasibility of implementing less intensive online mindfulness training for the RPg students. Participating in 90 minutes training of mindfulness over eight weeks effectively enhanced the resilience and wellbeing literacy and reduced psychological distress in this non-clinical sample of RPg students, which may potentially serve as an effective prevention method for mental illness.

In view of the non-significant findings of some subscales in trait mindfulness and compassion, a half-day or one-day silent retreat could be considered to integrate the existing FPFW curriculum to supplement the less intensive weekly practice. Future studies could further investigate the impact of dose-response relations in mindfulness program by including active control groups with a spectrum of intensity in training such as more traditional MBP (e.g., MBCT) or a less intensive mindfulness course with one day retreat to further examine dose specific changes in mindfulness training.

## Conclusion

The current RCT revealed the positive effects of an eight-week online MBP (i.e., FPFW) in alleviating emotional disturbances and enhancing trait mindfulness, resilience, and wellbeing literacy while no effects in compassion, sleep quality and subjective wellbeing. The positive effects persisted at the two months follow-up. The current study also empirically documents the improvement in wellbeing language use after mindfulness training and provides evidence that FPFW, a shorter and less intensive version of MBCT, effectively reduced mood-related symptoms and supported resilience among RPg students.

## Data Availability

The participants of this study did not give written consent for their data to be shared publicly. Further enquiries can be directed to the corresponding authors.

## Abbreviations

RCT: randomized controlled trial
RPg: Pursuing a research postgraduate
MBPs: mindfulness-based programs
FPFW: Finding Peace in a Frantic World
MBCT: Mindfulness-Based Cognitive Therapy
MBSR: Mindfulness Based Stress Reduction
CD-RISC2: Connor-Davidson Resilience Scale – 2 items
DASS-21: Depression, Anxiety and Stress Scale – 21 items
SOCS-O: Sussex-Oxford Compassion for Others Scale
SOCS-S: Sussex-Oxford Compassion for the Self Scale
FFMQ-15: 15-item Five Facet Mindfulness Questionnaire
ITT: intention-to-treat
